# Protective effects of DPP-4 inhibitor on podocyte injury in glomerular diseases

**DOI:** 10.1186/s12882-020-02060-9

**Published:** 2020-09-18

**Authors:** Ayano Kubo, Teruo Hidaka, Maiko Nakayama, Yu Sasaki, Miyuki Takagi, Hitoshi Suzuki, Yusuke Suzuki

**Affiliations:** 1 Department of Nephrology, Juntendo University Faculty of Medicine, 2-1-1 Hongo, Bunkyo-ku, Tokyo, 113-8421 Japan; 2 Department of Nephrology and Hypertension, Juntendo University Urayasu Hospital, 2-1-1 Tomioka, Urayasu-City, Chiba 279-0021 Japan

**Keywords:** DPP-4, Podocyte, Glomerular disease, Synaptopodin, Saxagliptin

## Abstract

**Background:**

Dipeptidyl peptidase-4 (DPP-4) is a serine protease that inhibits the degradation of glucagon-like peptide 1. DPP-4 inhibitors are used worldwide to treat type 2 diabetes mellitus and were recently shown to have pleiotropic effects such as anti-oxidant, anti-inflammatory, and anti-fibrotic actions. DPP-4 inhibitors improve albuminuria and renal injury including glomerular damage independent of its hypoglycemic effect. Although DPP-4 is mainly expressed in the kidney, the physiological function of DPP-4 remains unclear.

**Methods:**

The localization of renal DPP-4 activity was determined in human renal biopsy specimens with glycyl-1-prolyl-4-methoxy-2-naphthylamide and the effects of a DPP-4 inhibitor were examined in human cultured podocyte.

**Results:**

DPP-4 activity under normal conditions was observed in some Bowman’s capsular epithelial cells and proximal tubules, but not in the glomerulus. DPP-4 activity was observed in crescent formation in anti-neutrophil myeloperoxidase cytoplasmic antigen antibody nephritis, nodular lesions in diabetic nephropathy, and some podocytes in focal segmental glomerulosclerosis. Notably, the DPP-4 inhibitor saxagliptin suppressed DPP-4 activity in podocytes and the proximal tubules. To assess the effect of DPP-4 inhibitor on podocytes, human cultured podocytes were injured by Adriamycin, which increased DPP-4 activity; this activity was dose-dependently suppressed by saxagliptin. Treatment with saxagliptin maintained the structure of synaptopodin and RhoA. Saxagliptin also improved the detachment of podocytes.

**Conclusions:**

DPP-4 activity induces degradation of synaptopodin and reduction of RhoA, resulting in destruction of the podocyte cytoskeleton. Saxagliptin may have pleiotropic effects to prevent podocyte injury.

## Background

Dipeptidyl peptidase-4 (DPP-4) is a serine protease that exists in membrane-bound or soluble forms. The catalytic activity of DPP-4 removes the N-terminal dipeptide from peptides containing proline or alanine at the second position. The soluble form degrades glucagon-like peptide 1 (GLP-1), which is an incretin hormone secreted from the gastrointestinal tract in response to food intake. The active form of GLP-1 stimulates insulin secretion in a blood glucose-dependent manner. The active form of GLP-1 is rapidly degraded and inactivated by DPP-4. Therefore, DPP-4 inhibitors have hypoglycemic effects by inhibiting the degradation of GLP-1 in patients with type 2 diabetes mellitus [1]. However, the physiological role of DPP-4 remains unclear.

DPP-4 inhibitors were recently demonstrated to have pleiotropic effects such as anti-oxidant, anti-inflammatory, and anti-fibrotic actions [2]. Although DPP-4 is present throughout the body, its expression is high in the kidney [3]. According to a previous study, DPP-4 was expressed in mesangial cells [4], podocytes [5, 6] and tubules [5]. DPP-4 activity in the glomeruli shows different patterns between rat and human [6], but expression in humans is not completely understood.

DPP-4 activity is observed on renal proximal tubular cells and glomerular resident cells under non-disease conditions in rats [7]. In human, DPP-4 activity was observed in the glomeruli only under pathological renal conditions, but not in healthy kidneys [8]. DPP-4 activity has been implicated in kidney injuries and is negatively correlated with the estimated glomerular filtration rate [9]. Other studies found a correlation between increased DPP-4 activity and kidney diseases [10, 11]. Recent studies revealed that DPP-4 inhibitors protect against the progression of renal injuries, including glomerular damage independently of its hypoglycemic effects [12, 13]. The clinical SAVOR-TIMI53 trial demonstrated that the DPP-4 inhibitor saxagliptin significantly improves the albumin/creatinine ratio compared to placebo [14]. Those findings indicate that renal DPP-4 activity is involved in the pathogenesis of glomerular damage. However, the specific renal cells targeted by DPP-4 inhibition and mechanism of suppression of renal injury remain unclear. Human epithelial cells exhibit higher DPP-4 activity than that of other glomerular resident cells [15]. Furthermore, it is well-known that proteinuria and albuminuria are closely related to podocyte injury, suggesting that DPP-4 activity plays a key role in podocyte injury.

Adriamycin (ADR)-induced nephropathy is widely used as a podocyte injury model [16]. ADR induces thinning of the glomerular endothelium and podocyte effacement associated with loss of the size- and charge-specific barrier. In ADR mice model, DPP4 activity in the kidney and urinary nephrin loss were both increased, whereas GLP-1 concentrations were unchanged. Treatment with DPP-4 inhibitor significantly improved proteinuria, renal fibrosis and inflammation associated with improvement of urinary nephrin loss in ADR-treated mice. Moreover, DPP-4 inhibitor preserved the nephrin levels in cultured podocytes. Those findings suggest that activation of DPP-4 in the kidney has a role in the progression of renal disease, and DPP-4 inhibitor may prevent podocyte injury [17]. We predicted that DPP-4 activity induces podocyte injury, and thus saxagliptin may have renoprotective effects, particularly in podocyte injury. In the present study, we evaluated DPP-4 activity in the glomeruli in human kidney diseases. We also evaluated the correlation between podocytes and DPP-4 activity/inhibition in vitro using ADR-induced podocyte injury to examine the pathological roles of DPP-4 activity and its underlying mechanisms.

## Methods

### Human tissue samples

Renal biopsy samples were obtained from diagnostic renal biopsies performed at Juntendo University Hospital after the approval of the Ethics Committee on Human Research of Juntendo University Faculty of Medicine. Samples from human subjects with diabetic nephropathy (DN) with (*n* = 3) and without treatment with DPP-4 inhibitor (*n* = 9), minor glomerular abnormality (n = 3), focal segmental glomerular sclerosis (FSGS, *n* = 7), anti-neutrophil myeloperoxidase cytoplasmic antigen-antibody-related nephritis (ANCA-RN, *n* = 4), and nephrosclerosis (*n* = 1) were evaluated. Clinical data and treatment at the time of renal biopsy were shown in Table 1.

**Table 1:** Clinical data and treatment at the time of renal biopsy

	Diagnosis	Clinical data									Treatment							
		Gender	Blood pressure (mmHg)	sCr (mg/dL)	eGFR (mL/min/1.73m^2^)	Alb (g/dl)	Tchol (mg/dl)	HbA1c (%)	UPCR (g/gCr)	Duration of DM (years)	RAS inhibitor	DPP-4 inhibitor	Biguanide	SGLT2 inhibitor	SU	Glinide	αGI	Insulin
case 1	Minor glomerular abnormality^a^	female	90/52	0.55	102.0	4.2	210	5.3	0.0	0	0	0	0	0	0	0	0	0
case 2	Minor glomerular abnormality	female	100/63	0.50	115.3	4.6	170	5.4	0.5	0	0	0	0	0	0	0	0	0
case 3	Minor glomerular abnormality	female	110/64	0.85	62.8	4.3	156	6.0	0.1	0	0	0	0	0	0	0	0	0
case 4	ANCA-RN^a^	female	122/64	2.37	17.6	3.1	258	6.0	2.7	0	0	0	0	0	0	0	0	0
case 5	ANCA-RN	female	110/60	2.08	20.6	3.7	199	5.6	0.6	0	1	0	0	0	0	0	0	0
case 6	ANCA-RN with DPP-4i	male	125/60	1.77	32.8	3.4	170	6.6	1.7	15	1	1	1	0	1	0	0	0
case 7	ANCA-RN	female	160/70	0.98	42.8	3.8	210	6.7	0.4	2	1	0	0	0	0	0	0	0
case 8	FSGS^a^	female	120/82	1.04	50.7	3.0	224	5.2	1.6	0	1	0	0	0	0	0	0	0
case 9	FSGS	male	120/78	1.02	60.1	2.0	369	5.1	6.7	0	0	0	0	0	0	0	0	0
case 10	FSGS	male	142/70	0.91	72.1	1.9	379	5.7	5.3	0	0	0	0	0	0	0	0	0
case 11	FSGS	male	122/72	0.92	76.0	4.0	170	5.1	0.4	0	0	0	0	0	0	0	0	0
case 12	FSGS	male	128/74	1.94	33.6	4.7	199	5.5	0.1	0	0	0	0	0	0	0	0	0
case 13	FSGS	female	122/62	1.03	48.3	4.0	217	5.3	2.8	0	1	0	0	0	0	0	0	0
case 14	FSGS	female	116/60	0.81	69.2	2.9	216	5.9	7.3	0	0	0	0	0	0	0	0	0
case 15	Nephrosclerosis^a^	male	122/78	0.75	76.3	4.1	218	5.9	0.0	0	1	0	0	0	0	0	0	0
case 16	DN w/o DPP-4i^ab^	male	118/62	0.85	65.7	4.4	210	6.7	0.9	unknown	1	0	0	0	0	0	0	0
case 17	DN w/o DPP-4i^b^	male	132/78	1.50	48.4	3.4	208	6.4	5.0	20	1	0	0	1	0	0	0	1
case 18	DN w/o DPP-4i^b^	male	134/84	0.77	71.2	2.1	227	8.1	5.5	13	1	0	0	0	0	0	0	1
case 19	DN w/o DPP-4i^b^	female	140/80	0.36	137.1	2.6	267	12.7	3.2	unknown	0	1	1	0	0	0	0	0
case 20	DN with DPP-4i	male	162/66	2.21	24.2	2.4	274	7.1	4.5	27	1	1	0	0	1	1	1	0
case 21	DN with DPP-4i	male	138/76	2.00	32.7	2.4	245	9.3	6.3	4	0	1	0	0	0	0	0	0
case 22	DN with DPP-4i	male	130/70	2.00	29.2	3.9	150	6.4	7.3	3	1	1	0	0	0	0	0	0
case 23	DN with DPP-4i	male	138/68	1.82	29.3	4.4	162	6.0	0.7	30	1	1	1	0	1	0	0	0
case 24	DN with DPP-4i	male	152/78	0.76	74.8	3.9	184	7.0	0.2	10	1	1	0	1	1	0	0	0
case 25	DN with DPP-4i^b^	male	132/68	2.07	27.9	3.7	127	6.8	3.3	5	0	1	1	0	1	0	0	0
case 26	DN with DPP-4i^b^	male	112/82	0.58	106.0	4.1	564	8.1	2.8	6	1	1	1	1	0	0	0	1
case 27	DN with DPP-4i^b^	male	148/64	1.46	32.9	2.3	478	8.5	13.5	12	0	1	0	0	0	0	0	0

*ANCA-RN* ANCA-related nephritis, *FSGS* focal segmental glomerular sclerosis, *DN w/o DPP-4i* diabetic nephropathy without DPP-4 inhibitor treatment, *DN with DPP-4i* diabetic nephropathy treated with DPP-4 inhibitor, *sCr* serum creatinine, *eGFR* estimemated glomerular filtration rate, *Alb* serum albumin, *Tchol* serum total cholesterol, *UPCR* urinary protein excretion, *DM* diabetes mellitus, *RAS inhibitor* renin−angiotensin system inhibitor, *SU* sulfonylurea, *αGI* α-glucosidase inhibitor, *SGLT2* sodium glucose cotransporter 2

0: without treatment, 1:with treatment

**Fig. 1:**
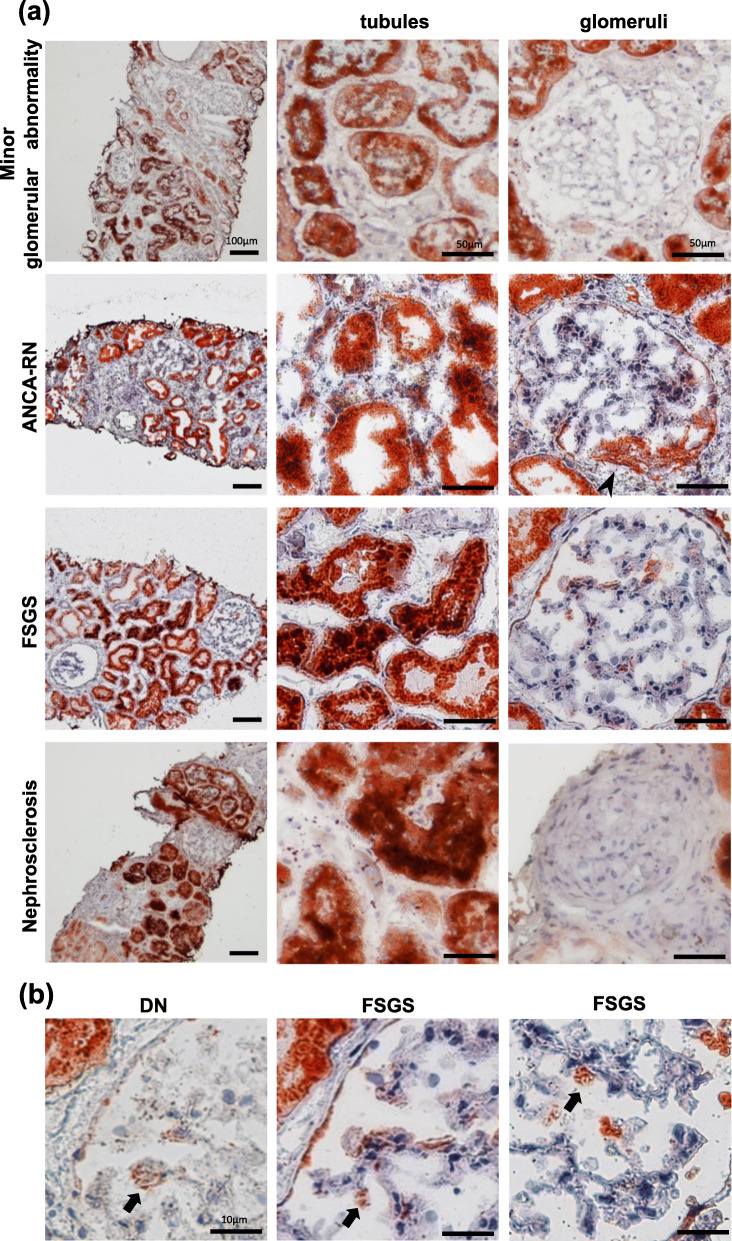
DPP-4-active lesions in glomerular diseases. **a** In minor glomerular abnormalities, DPP-4 activity was observed in the parietal cells and proximal tubules. In cases with ANCA-RN, DPP-4 activity was observed in crescent formation (arrow head). In cases with nephrosclerosis, DPP-4 activity was not observed in the glomeruli. **b** In other patients with DN and FSGS, DPP-4 activity was detected in podocytes (arrow)

**Fig. 2:**
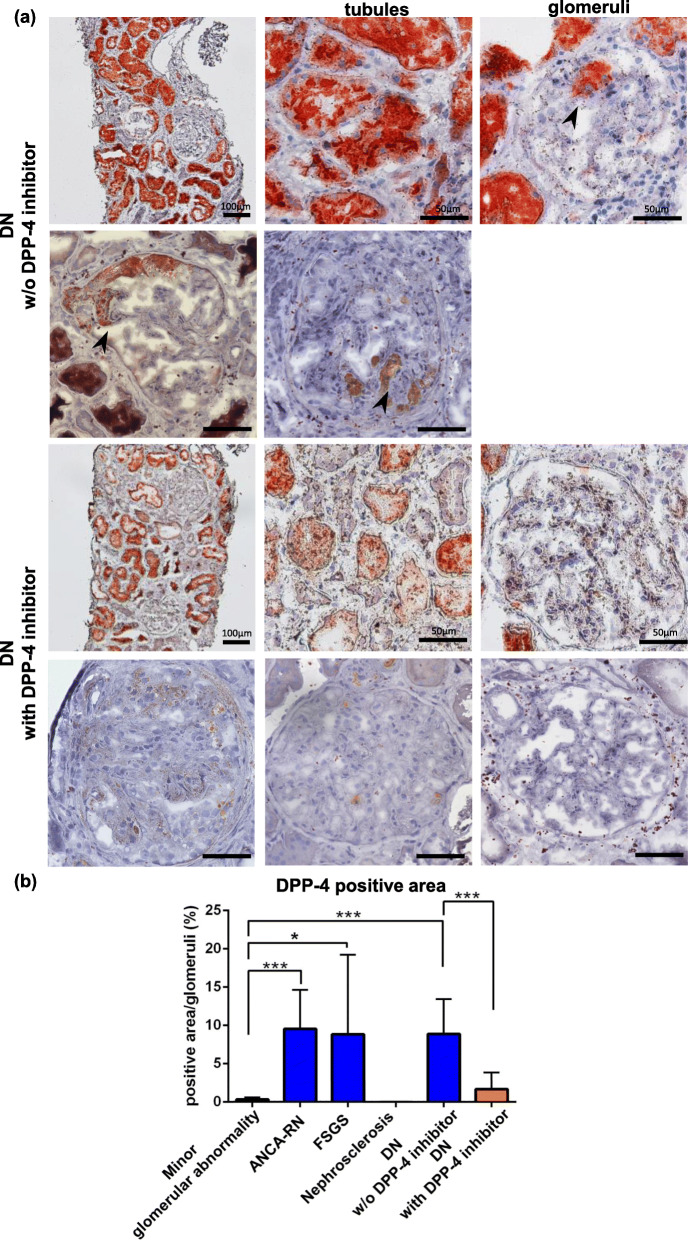
DPP-4 activity in DN with or without DPP-4 inhibitor. **a** Partial podocytes, nodular lesion (arrow head), and proximal tubules were stained with DPP-4 in a patient with DN without DPP-4 inhibitor treatment (w/o DPP-4 inhibitor). Renal DPP-4 activity was suppressed by DPP-4 inhibitor, compared to the case not treated with DPP-4 inhibitor. **b** DPP-4 positive area was significantly high in FSGS, ANCA-RN and DN w/o DPP-4 inhibitor treatment, compared to minor glomerular abnormality. DPP-4 activity in the glomeruli was significantly suppressed by treatment with DPP-4 inhibitor. *: *P* < 0.05, ***: *P* < 0.001

^a^: indicated in Fig. 1, ^b^: indicated in Fig. 2

### Assessment of DPP-4 activity in renal biopsy specimens

Frozen kidney sections (3 μm) were fixed with formalin, phosphate-buffered saline (PBS), and acetone (1:35:15) and washed with water. The samples were incubated with a coloring solution (1.76 mol/L glycyl-prolyl-4-methoxy-β-naphthylamide, 2.52 mol/L Fast Blue B, 3.71 vol% *N*, *N*-dimethyl formamide, 95.7 mmol/L phosphate buffer) [5]. After rinsing with water, images were acquired with a BX43 Microscope (Olympus, Tokyo, Japan). Distributions of DPP-4 activity in the kidney and semi-quantitative DPP-4 activity were shown in Table 2. We quantified DPP-4 positive area / glomeruli (%) using open-source software ImageJ [18] (Fig. 2b).

**Table 2:** Distributions of DPP-4 activity in kidney

	Diagnosis	Podocyte	Bowman’s capsule	Proximal tubule	Distal tubule	Interstitial tissue	others
case 1	Minor glomerular abnormality^a^	–	+/−	+	–	–	
case 2	Minor glomerular abnormality	–	+/−	++	–	–	
case 3	Minor glomerular abnormality	–	+	++	–	–	
case 4	ANCA-RN^a^	–	+	++	–	–	crescent ++
case 5	ANCA-RN	–	+	+	–	–	crescent +
case 6	ANCA-RN with DPP-4i	–	–	+	–	–	
case 7	ANCA-RN	–	+/−	+	–	–	crescent -
case 8	FSGS^a^	+	+	++	–	–	
case 9	FSGS	+	+	++	–	–	
case 10	FSGS	+	+	++	–	–	
case 11	FSGS	+/−	+	++	–	–	
case 12	FSGS	+	+/−	+	–	–	
case 13	FSGS	+	+/−	++	–	–	
case 14	FSGS	+	+	++	–	–	
case 15	Nephrosclerosis^a^	–	–	++	–	–	
case 16	DN w/o DPP-4i^ab^	+	+/−	++	–	–	nodule +
case 17	DN w/o DPP-4i^b^	+	+/−	+	–	–	nodule +
case 18	DN w/o DPP-4i^b^	+/−	+	++	–	–	nodule +
case 19	DN with DPP-4i^b^	+/−	+/−	+	–	–	
case 20	DN with DPP-4i	+	+	++	–	–	
case 21	DN with DPP-4i	+/−	+/−	+	–	–	
case 22	DN with DPP-4i	+/−	+/−	+	–	–	
case 23	DN with DPP-4i	–	–	+	–	–	
case 24	DN with DPP-4i	+	+/−	+	–	–	
case 25	DN with DPP-4i^b^	–	+	–	–	–	
case 26	DN with DPP-4i^b^	–	–	+/−	–	–	
case 27	DN with DPP-4i^b^	+	+/−	+/−	–	–	nodule +

*ANCA-RN* ANCA-related nephritis, *FSGS* focal segmental glomerular sclerosis, *DN w/o DPP-4i* diabetic nephropathy without DPP-4 inhibitor treatment, *DN with DPP-4i* diabetic nephropathy treated with DPP-4 inhibitor

DPP-4 activity was evaluated in -, +/−, +, ++

^a^: indicated in Fig. 1, ^b^: indicated in Fig. 2

### Cell cultures and measurement of DPP4 activity

Conditionally immortalized human podocytes were kindly provided by Dr. Moin A. Saleem (Bristol Royal Hospital for Children Bristol, Bristol, UK) and cultured as previously described [19]. Cultured podocytes were treated with 0.15 μg/mL of ADR (ADR group) or normal saline (control) for 10 days. At 2 and 4 days after ADR treatment, podocytes were treated with saxagliptin (1, 10, and 100 nM) (ADR + saxagliptin group). After treatment, podocytes were collected using a scraper, pelleted by centrifugation, and washed twice with ice-cold PBS. DPP-4 activities of cultured podocytes were measured using DPP4 Activity Assay Kit (Abcam, Cambridge, UK) and FlexStation 3 Multi-Mode Microplate Reader (Molecular Devices, Sunnyvale, CA, USA) according to the manufacturer’s protocol.

### Immunostaining for cytoskeleton protein

Differentiated podocytes were cultured on collagen type I-coated cover slips and then treated with saline (control), ADR alone (ADR group), or ADR with saxagliptin (ADR + saxagliptin group) for 8 days. The cells were fixed with 2% paraformaldehyde and incubated with blocking solution (2% fetal calf serum, 2% bovine serum albumin, 0.2% fish gelatin in PBS). The primary antibody against synaptopodin (Progen Biotechnik GmbH, Heidelberg, Germany) was used at a 1:10 dilution. The primary antibody against Alexa Fluor 555 Phalloidin (Thermo Fisher Scientific, Waltham, MA, USA) as a marker of F-actin, a stress fiber, was used at 1:250 dilution. As a secondary antibody, Alexa Fluor 488 goat anti-mouse IgG (Thermo Fisher Scientific) was used at a 1:300 dilution. Images were acquired using FV1000 Confocal Microscope (Olympus). The areas of synaptopodin and F-actin were measured with open-source software ImageJ [18].

### Assessment of podocyte detachment

Podocytes were cultured in 6-well plates at a concentration of 1.5 × 10^5^ cells/well. On day 0, the number of cells per field were counted as a baseline number. The average number of cells in 5 fields in 3 independent sets of experiments was determined. After treatment with ADR with or without 100 nM saxagliptin for 2 days, podocytes were counted in the same 5 fields of each well. The ratio of detachment was also evaluated.

### Western blotting

The cell pellet was re-suspended in 3-[(3-cholamidopropyl) dimethylammonio]-1-propanesulfonate buffer and incubated on ice for 30 min. The cell lysate was cleared by centrifugation for 10 min. Samples were separated by sodium dodecyl sulfate–polyacrylamide gels and then proteins were transferred to membranes and blocked with Block-ACE (DS Pharma Biomedical Co., Ltd., Osaka, Japan). The membranes were incubated with the appropriate primary antibodies. The antibodies against synaptopodin (Santa Cruz Biotechnology, Inc., Dallas, TX, USA) and RhoA (Santa Cruz Biotechnology, Inc., Dallas, TX, USA) were used at 1:500 and 1:300 dilutions, respectively. Peroxidase-conjugated goat anti-mouse IgG was used as a secondary antibody at a 1:10,000 dilution (Jackson Immunoresearch, West Grove, PA, USA). Equal protein loading was confirmed by reprobing the membrane with GAPDH at 1: 20,000 (Sigma-Aldrich, St. Louis, MO, USA). Images were scanned with a C-Digit chemiluminescent western blot scanner, and densitometry analysis was performed using Image Studio Digits software (LI-COR Biosciences, Lincoln, NE, USA).

### Statistics

All values are shown as the means ± standard deviation. Statistical significance (defined as *P* < 0.05) was calculated using Prism 6.0 software (GraphPad, Inc., La Jolla, CA, USA) followed by *t*-test.

## Results

### DPP-4 active lesions in glomerular diseases

DPP-4 was clearly detected in the proximal tubules and Bowman’s capsule, but not in the distal tubules and interstitial tissues of any glomerular disease (Table 2). Non-immune glomerular diseases including minor glomerular abnormality and nephrosclerosis showed no clear DPP-4 active lesion in glomerular resident cells (Fig. 1a, Additional file 1). Whereas, DPP-4 active lesions were detected in some podocytes in DN w/o DPP-4 inhibitor treatment and FSGS (Fig. 1a, b, Additional file 1). Furthermore, DPP-4 activity was detected in nodular lesions as Kimmelstiel-Wilson lesions in DN and fibrocellular crescents in ANCA-RN (Fig. 1a, Additional file 1). Quantitative analysis of DPP-4 positive area was significantly high in FSGS, ANCA-RN and DN w/o DPP-4 inhibitor treatment, compared to minor glomerular abnormality (FSGS; *P* < 0.05, ANCA-RN; *P* < 0.001, DN w/o DPP-4 inhibitor treatment; P < 0.001) (Fig. 2b).

### DPP-4 inhibitor could suppress renal DPP-4 activity in diabetic nephropathy

We next evaluated renal DPP-4 activity in cases treated with DPP-4 inhibitor. DPP-4 activity in the glomeruli was significantly suppressed by DPP-4 inhibitor, compared to in case not treated with DPP-4 inhibitor (P < 0.001) (Fig. 2a, b).

### In human cultured podocytes, DPP-4 activity was suppressed by DPP-4 inhibitor

**Fig. 3:**
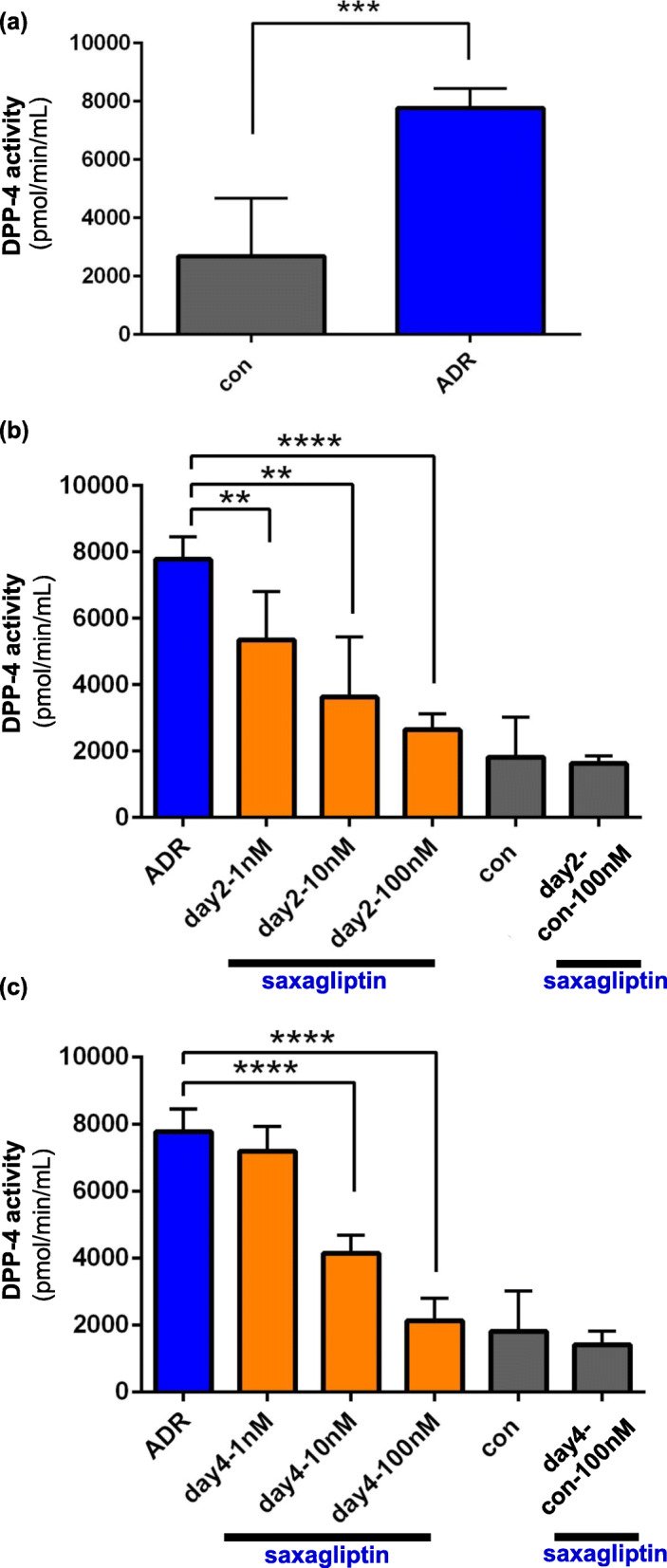
DPP-4 activity in human cultured podocytes with or without ADR treatment. **a** DPP-4 activity in injured podocytes was significantly higher than that in controls. **b**, **c** DPP-4 activity in podocytes using saxagliptin (100 nM) was significantly lower than that in podocytes using 1 nM saxagliptin at days 2 and 4. DPP-4 activity was not significantly reduced in control podocytes by treatment with saxagliptin. **: *P* < 0.01, ***: P < 0.001, ****: *P* < 0.0001

DPP-4 activity in cultured podocytes with or without DPP-4 inhibitor was examined. We found that ADR significantly induced DPP-4 activation in podocytes (control; 2682 ± 1994, ADR; 7774 ± 669.2 pmol/min/L, *P* < 0.01) (Fig. 3a). In the ADR + saxagliptin group at day 2, saxagliptin significantly suppressed ADR-induced DPP-4 activation in podocytes in a dose-dependent manner (1 nM; 5343 ± 1448,10 nM; 3621 ± 1806, 100 nM; 2638 ± 473.6, ADR vs. saxagliptin 1 nM; *p* = 0.0093, ADR vs. 10 nM; P < 0.01, ADR vs. 100 nM; *P* < 0.0001) (Fig. 3b). Even at day 4 in the ADR + saxagliptin group, saxagliptin continued to suppress DPP-4 activation in injured podocytes (1 nM; 7190 ± 748.9, 10 nM; 4146 ± 536.1, 100 nM; 2127 ± 666.5, ADR vs. saxagliptin 10 nM; P < 0.0001, ADR vs. 100 nM; P < 0.0001) (Fig. 3c). Meanwhile, DPP-4 activity was not suppressed by saxagliptin in control podocytes (Fig. 3b, c).

### DPP-4 inhibitor prevents functional deterioration of podocytes through maintenance of cytoskeleton-associated proteins

**Fig. 4:**
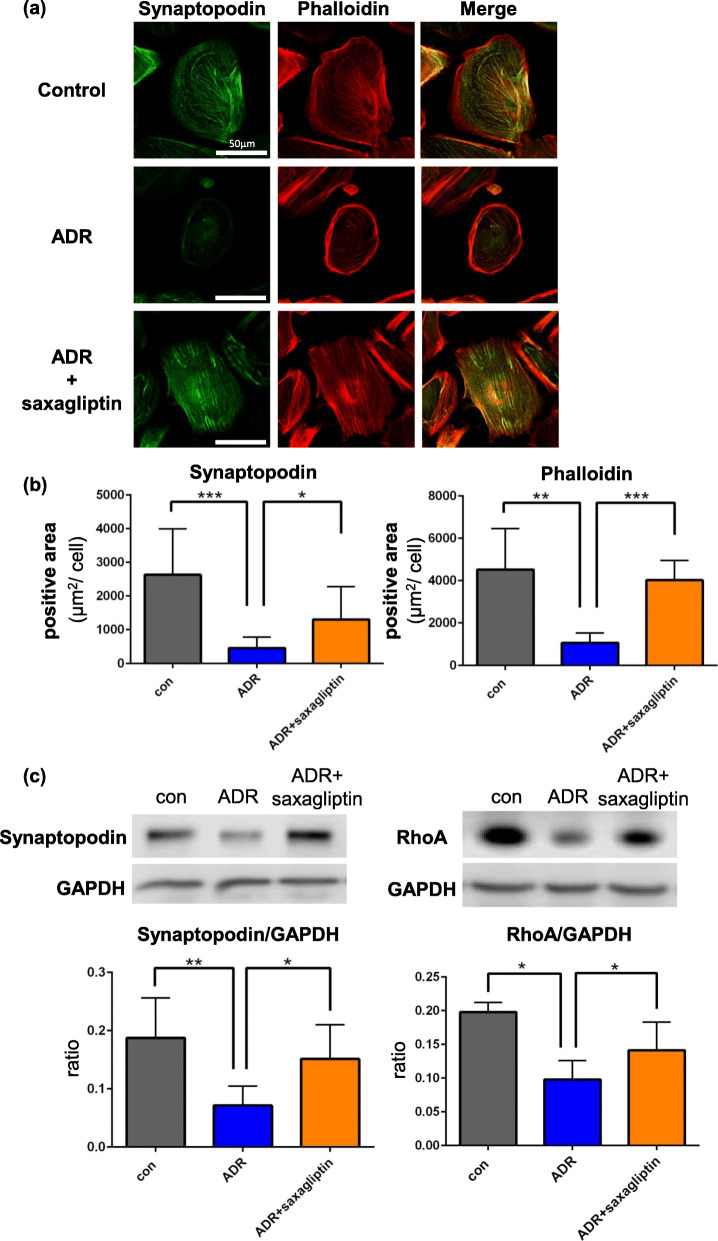
Cytoskeleton-associated proteins were protected by saxagliptin after podocyte injuries by ADR. **a** In the control and ADR + saxagliptin group, synaptopodin and stress fibers were observed in the cytoplasm. In the ADR group, synaptopodin and stress fibers were not observed in the cytoplasm. **b** Area of synaptopodin/cell and stress fibers/cell in the saxagliptin group was maintained compared to that in the ADR group. **c** Levels of synaptopodin and RhoA in the ADR group was significantly lower than that in the control and ADR + saxagliptin group. *: P < 0.05, ***: P < 0.001

We examined whether ADR-induced podocyte injury is related to DPP-4-dependent integrity of cytoskeleton-associated proteins such as synaptopodin, F-actin, and stress fibers. Synaptopodin and stress fibers were not observed in the cytoplasm of the ADR group (Fig. 4a). Positive areas of synaptopodin in podocytes of the ADR group showed significant shrinkage compared to the control (control; 2632 ± 1366, ADR; 461 ± 328, control vs. ADR; *P* < 0.001). Stress fibers also showed significant shrinkage (control; 4522 ± 1933, ADR; 1070 ± 451, control vs. ADR; *P* < 0.01). In both the control and ADR + saxagliptin groups, synaptopodin and stress fibers were observed in the cytoplasm. In the ADR + saxagliptin group, the areas of synaptopodin (1297 ± 982) and stress fibers (4021 ± 931) were clearly maintained compared to those in the ADR group (synaptopodin; *P* < 0.05, stress fiber; *P* < 0.001) (Fig. 4b).

ADR treatment resulted in significant degradation of synaptopodin, which was significantly rescued by saxagliptin (control vs. ADR; *P* < 0.01, ADR vs. ADR + saxagliptin; P < 0.05). RhoA was also maintained in the saxagliptin treatment group (control vs. ADR; P < 0.05, ADR vs. ADR + saxagliptin; P < 0.05) (Fig. 4c). Original images were shown in Additional file 2.

**Fig. 5:**
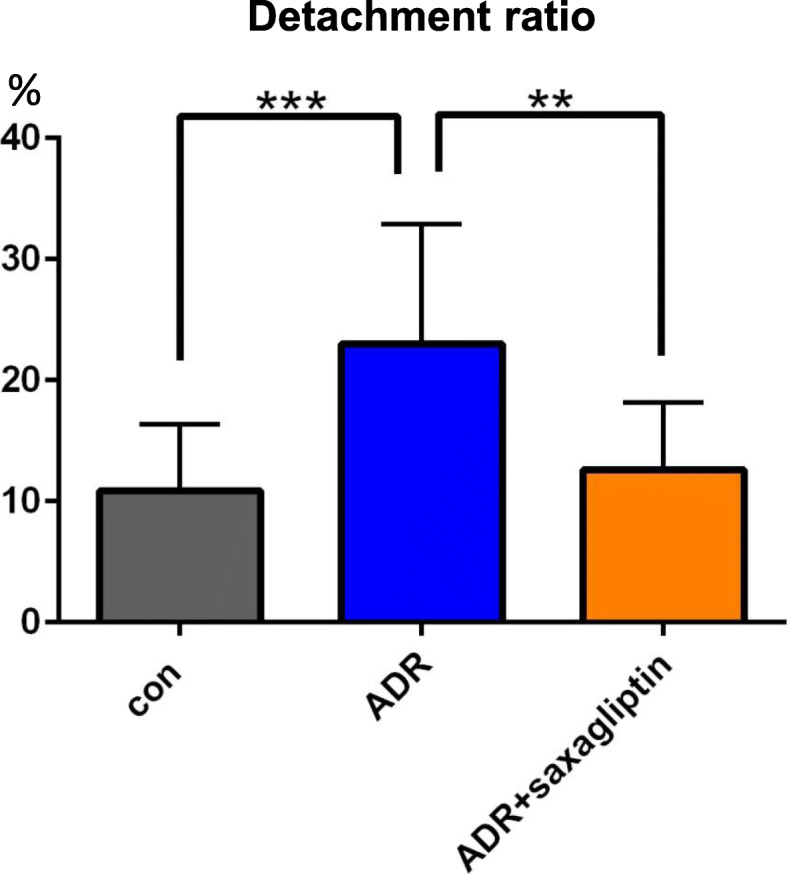
Saxagliptin prevents podocyte detachment. In the ADR + saxagliptin group, the podocyte detachment ratio was significantly suppressed compared to that in the ADR group. *: P < 0.05

To examine whether endogenous DPP-4-dependent injury in podocytes induces functional deterioration, a detachment assay of podocytes was performed. In the control, the detachment was observed in 10.86 ± 5.50% of podocytes after 48 h. ADR treatment significantly induced the cell detachment (23.00 ± 9.89%, P < 0.001). However, podocyte detachment was significantly improved by saxagliptin (12.62 ± 5.53%, P < 0.01) (Fig. 5).

## Discussion

The present study demonstrated that glomerular DPP-4 is activated in glomerular diseases, such as DN, FSGS, and ANCA-RN. Particularly, DPP-4 activity was related to podocyte injury with decreased expression of cytoskeleton proteins. Notably, DPP-4 inhibitor improved the podocyte injury in vitro and DPP-4 activity following the treatment was suppressed in DN.

DPP-4 is a conserved exopeptidase with protein regulatory activities. DPP-4 can either be anchored to the plasma membrane as a homodimeric type II transmembrane glycoprotein [20] or circulate in the extracellular compartment. Thus, DPP-4 affects systemic physiological functions such as glucose metabolism, cellular signaling, and oxidative stress, suggesting that renal DPP-4 activity may be involved in the progression of kidney diseases. Diverse effects of DPP-4 inhibitors have been examined in animal models of both diabetic and nondiabetic CKD [5, 12, 21]. DPP-4 inhibitor had beneficial renal effects such as suppression of urinary albumin excretion, preventing mesangial matrix expansion [22], reduction interstitial fibrosis and podocyte loss [23]. Those effects are independent of GLP-1/GLP1R pathway. In fact, DPP-4 inhibitors downregulated advanced glycation end product (AGE)-Receptor of AGE axis, oxidative stress, lymphocyte infiltration and intercellular adhesion molecule-1 (ICAM-1) mRNA levels independent of blood pressure and plasma glucose [24]. The proteomic analysis showed potential contribution of DPP-4 inhibitor in signaling pathways, such as collagen I homeostasis, HNRNPA1, YB-1, thymosin β4 and TGF-β1, which may explain the mechanism of anti-fibrosis of DPP-4 inhibitor [25]. Thus, there are obviously different pathways involved in the renoprotective properties of DPP-4 inhibitors.

In the present study, glomerular DPP-4 activity was enhanced in glomerular diseases, while basal DPP-4 expression on parietal epithelial cells in Bowman’s capsules and proximal tubules was detected. In fact, DPP-4 activity was observed in nodular lesions in DN and crescentic lesions in ANCA-RN. Crescent formation is known to be related to podocyte injury/detachment. The formation of tight junctions between podocytes may be an early ultrastructural alteration in crescent formation, preceding foot process effacement and podocyte bridge formation in response to inflammatory injury [26]. The tumor suppressor protein p53, a transcription factor regulated by phosphorylation, increases the expression of genes that control growth arrest or cell death. Knockdown of p53 inhibited mitochondrial dysfunction and subsequent podocyte apoptosis in aldosterone-induced podocyte injury [27], while p53 is also involved in suppressing cell ferroptosis by directly inhibiting DPP4 activity. Interestingly, DPP-4 activity was enhanced in crescentic lesions in the human kidneys. Podocyte loss contributes to progressive sclerosis in association with Kimmelstiel-Wilson nodule formation in DN through vascular endothelial growth factor (VEGF)-A and enhanced nitric oxide synthase deficiency [28]. Because the angiogenic effects of DPP-4 are partly due to VEGF receptor signaling [29], DPP-4 may have direct therapeutic effects on nodular lesions in DN beyond glucose control.

In addition to nodular lesion and crescentic formation, DPP-4 activity was observed in podocytes in some cases of DN and FSGS. In vitro*,* we clarified that ADR-induced podocyte injury increases DPP-4 activity and decreases the expression of RhoA and cytoskeleton-associated proteins, such as synaptopodin, which was directly rescued by the DPP-4 inhibitor saxagliptin. Maintenance of synaptopodin resulted maintained cell formation through RhoA signaling. RhoA is a family of small GTPases, which controls signal-transduction pathways that influence various aspects of cell behavior, including cytoskeletal dynamics [30]. The possible effect of DPP-4 inhibitor is preventing apoptosis pathway via RhoA. DPP-4 inhibitor normalized podocyte apoptosis in the kidneys of db/db mice [31, 32]. The Yes-associated protein (YAP) is a major downstream cascade of the Hippo pathway and is known to inhibit dendrin mediated apoptosis in podocytes. Huang et al. reported that RhoA activation of YAP could inhibit apoptosis of podocyte through dendrin, and clarified the connection among RhoA, mammalian diaphanous-related formins (mDia; downstream effector of RhoA), YAP and dendrin in podocyte. Knocking down dendrin expression with significantly abolished RhoA, mDia or YAP deficiency induced podocyte apoptosis. Thus, those data demonstrate that RhoA/mDia/YAP deficiency induced podocyte apoptosis [33]. They also found that RhoA expression was significantly decreased in ADR-injured podocytes [33]. Another study indicated that RhoA play a critical role in DN by mediating the podocyte apoptosis through YAP [34]. Li et al. reported that DPP-4 inhibitor restored RhoA level [35]. Thus, we discussed that saxagliptin may prevent RhoA expression and podocyte detachment through RhoA/YAP pathway.

Meanwhile, Meliambro K et al. found increased expression of phosphorylated YAP protein in glomeruli of patients with FSGS [36]. RhoA may be a novel molecular target for DPP-4 inhibitor. Furthermore, saxagliptin may restore RhoA level through preventing degradation of synaptopodin. Synaptopodin is a novel regulator of RhoA signaling and induces stress fibers by competitively blocking ubiquitination of RhoA [37]. Stress fiber production is necessary for rearrangement of the podocyte actin cytoskeleton. This morphological change is a type of adaptation that enables damaged podocytes to bind to the glomerular basement membrane and maintain the glomeruli structure [38]. Loss of synaptopodin in podocytes causes the loss of stress fibers and formation of aberrant non-polarized filopodia, which suppresses rearrangement of the podocyte actin cytoskeleton [37]. Ilatovskaya et al. found that reactive oxygen species production promotes podocyte injury by enhancing calcium influx via canonical transient receptor potential channel (TRPC) [39], which is present on the surface of podocytes and activates calcineurin. Calcineurin induces dephosphorylation of synaptopodin, resulting in degradation of synaptopodin by cathepsin L [40]. ADR/doxorubicin, which was used in the present study, is known to induce podocyte toxicity by reactive oxygen species via NADPH-CYP reductase. Therefore, local oxidative stress may induce DPP-4 activity leading to podocyte damage through activating TRPC/calcineurin cascade. One of the DPP-4 inhibitors, saxagliptin, may inhibit TRPC/calcineurin cascade, which results in protection of synaptopodin.

Podocytes are important cells in the barrier function of the glomerular filter and their functions are largely based on the cell architecture. Podocyte loss underlies the progression of glomerulosclerosis in animal model and human glomerular diseases [41, 42]. The major cause of podocyte loss appears to be detachment from the glomerular basement membrane (GBM), leading to bare GBM and tuft adhesion to the Bowman’s capsule, leading to glomerulosclerosis [43]. Maintaining the cytoskeleton is critical for preventing cell detachment. The detachment assay clearly indicated that saxagliptin suppressed detachment in cultured podocytes treated with ADR. These results indicate that saxagliptin maintains the cytoskeleton of podocytes to prevent the progression to glomerular sclerosis. Additionally, podocytes may be the target cells of DPP-4 inhibitors and targets for therapeutic applications of saxagliptin for some glomerular diseases.

There were some limitations in the present study. First, whether DPP-4 activity was decreased by treatment with saxagliptin was still unclear because of the small sample size. Second, the renoprotective effect must be examined using other DPP-4 inhibitors. Third, DPP-4 activity is also observed in endothelial cells and mesangial cells in the glomeruli. However, we only evaluated human podocytes in this study. Further studies are needed to evaluate other types of glomerular resident cells. For accurate identification of the pathway of DPP-4 inhibitor, RNA sequencing, miRNA profiling, and proteomic analysis are preferable. It is our future task to identify the accurate renoprotective pathway of DPP-4 inhibitor.

This is the first report that DPP-4 activity was increased in human various kidney diseases by in situ staining. Our future goal is to expand the clinical application of DPP-4 inhibitors to other kidney diseases in addition to DN. We think that in situ staining is low-cost and reliable staining method.

## Conclusion

The present study revealed that DPP-4 activity was increased in human podocytes of glomerular diseases. Saxagliptin significantly suppressed DPP-4 activity and prevented the degradation of synaptopodin and the cellular detachment. DPP-4 inhibitors may be useful for developing treatments for glomerular disease with podocyte injury.

## Supplementary information

**Additional file 1.** DPP-4-active lesions in human several glomerular diseases. In addition to Figs. 1 and 2, glomerular DPP-4 staining was shown in several kidney diseases. In cases with ANCA-RN, DPP-4 activity was observed in crescent formation (arrow head). In other patients with DN and FSGS, DPP-4 activity was detected in podocytes (arrow). Scale bar: 50 μm.

**Additional file 2.** Original western blot images. (a) The blot of synaptopodin and GAPDH shown in Fig. 4c were indicated by red square. (b) The blot of RhoA and GAPDH shown in Fig. 4c were indicated by red square.

## Data Availability

The datasets used and/or analyzed during the current study are available from the corresponding author on reasonable request. The datasets supporting the conclusions of this article are included within the article.

## Abbreviation

DPP-4: Dipeptidyl peptidase-4
GLP-1: Glucagon-like peptide
ADR: Adriamycin
DN: Diabetic nephropathy
FSGS: Focal segmental glomerular sclerosis
ANCA-RN: Anti-neutrophil myeloperoxidase cytoplasmic antigen-antibody-related nephritis
PBS: Phosphate-buffered saline
VEGF: Vascular endothelial growth factor
GBM: Glomerular basement membrane
AGE: Advanced glycation end product
ICAM-1: Intercellular adhesion molecule-1
YAP: Yes-associated protein
TRPC: Transient receptor potential channel
