# Supplementary figures and images for: Protective effects of DPP-4 inhibitor on podocyte injury in glomerular diseases
Source: BMC Nephrol. 2020 Sep 18;21:402. doi: 10.1186/s12882-020-02060-9 (PMC7501714; doi:10.1186/s12882-020-02060-9)

## Slide 1
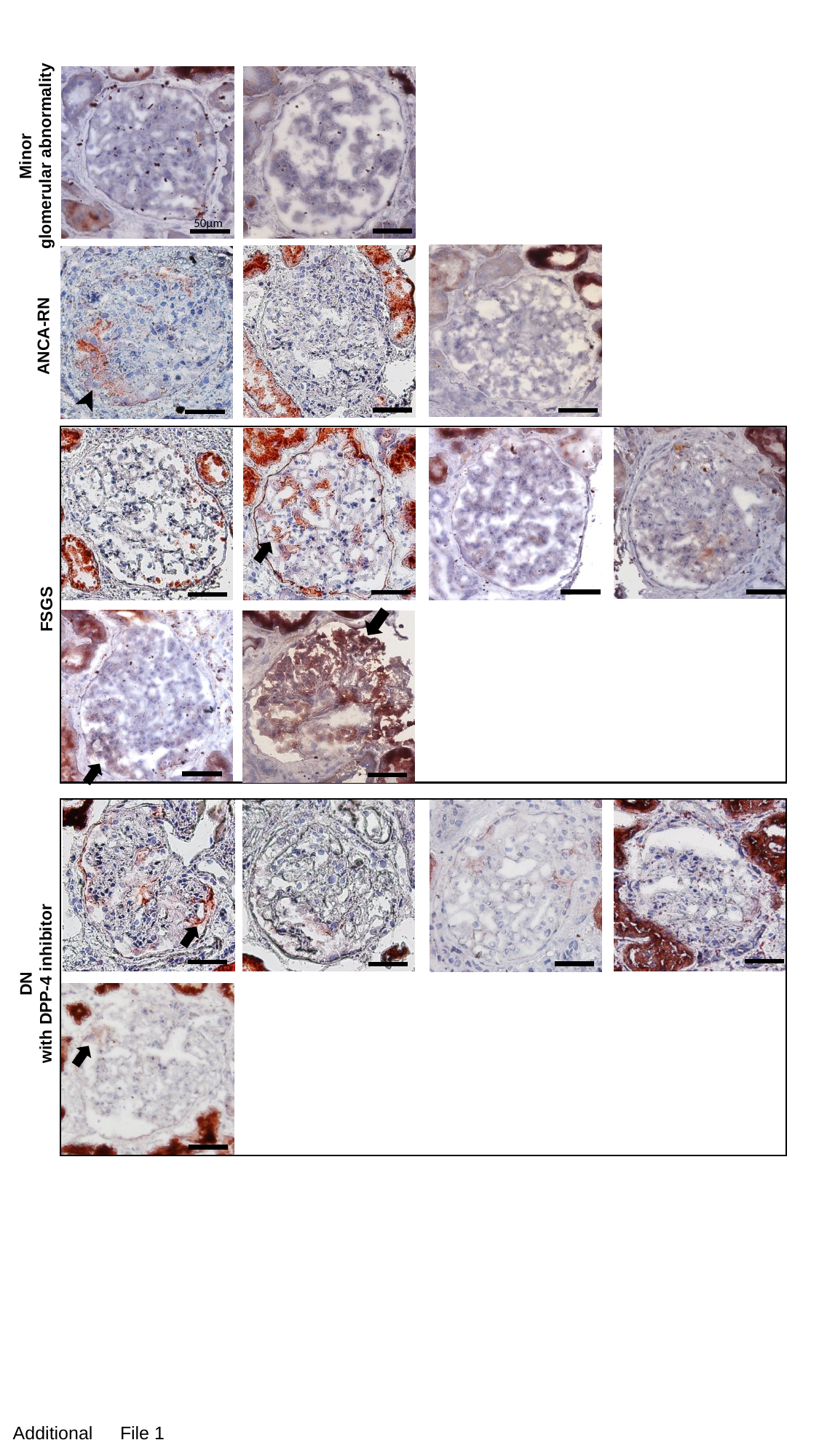

Minor
glomerular abnormality
50µm
ANCA-RN
➤
FSGS
DN
with DPP-4 inhibitor
Additional　File 1

Supplement: Supplementary file 1 — Additional file 1. DPP-4-active lesions in human several glomerular diseases. In addition to Figs. 1 and 2, glomerular DPP-4 staining was shown in several kidney diseases. In cases with ANCA-RN, DPP-4 activity was observed in crescent formation (arrow head). In other patients with DN and FSGS, DPP-4 activity was detected in podocytes (arrow). Scale bar: 50 μm. [file 12882_2020_2060_MOESM1_ESM.pptx]
