# Supplementary material for: Protective effects of DPP-4 inhibitor on podocyte injury in glomerular diseases
Source: BMC Nephrol. 2020 Sep 18;21:402. doi: 10.1186/s12882-020-02060-9 (PMC7501714; doi:10.1186/s12882-020-02060-9)
Supplement: Supplementary file 2 — Additional file 2. Original western blot images. (a) The blot of synaptopodin and GAPDH shown in Fig. 4c were indicated by red square. (b) The blot of RhoA and GAPDH shown in Fig. 4c were indicated by red square. [file 12882_2020_2060_MOESM2_ESM.pptx]

## Slide 1
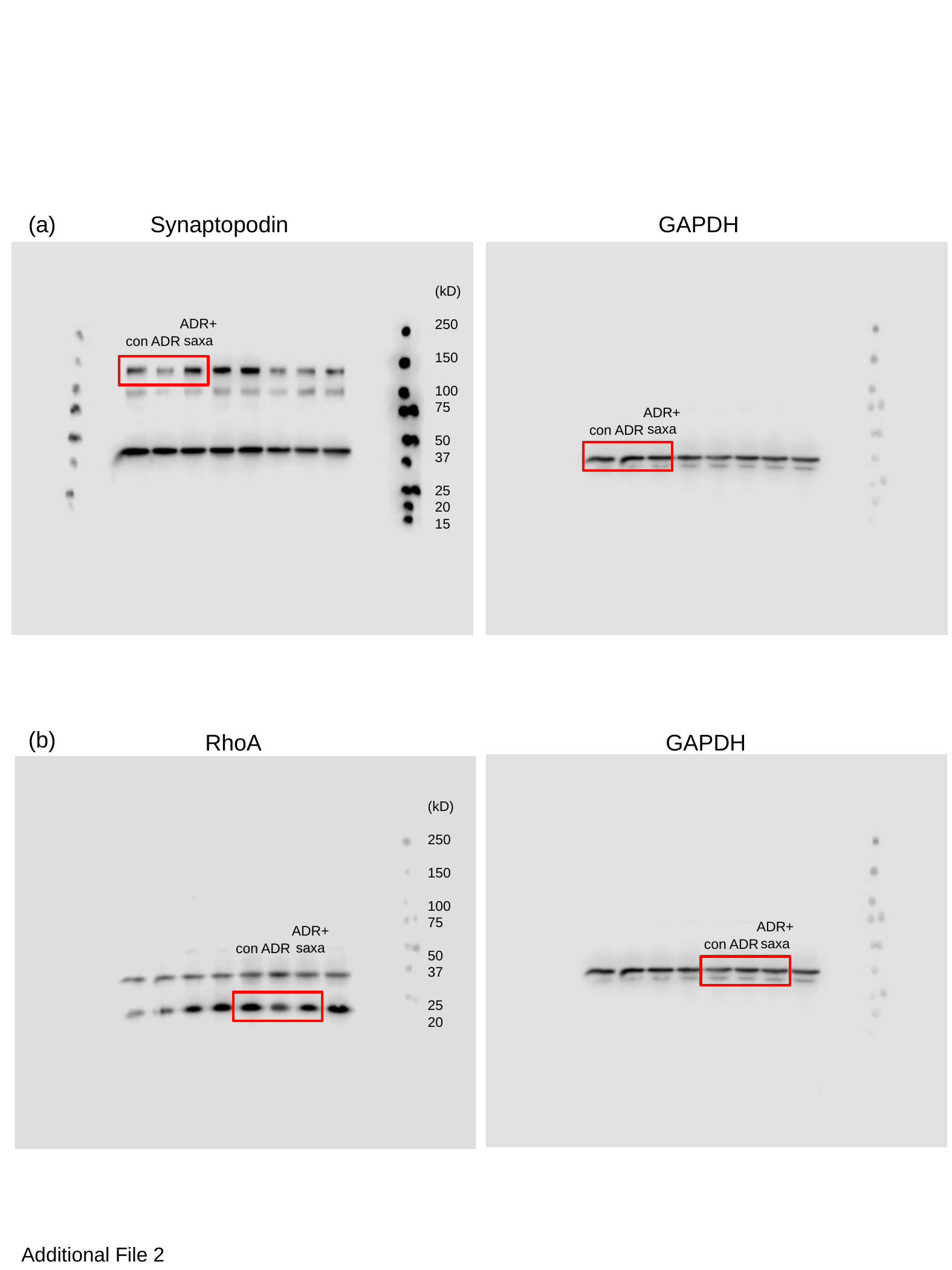

(a)
Synaptopodin
GAPDH
(kD)
250
150
100
75
50
37
25
20
15
ADR+
saxa
con ADR
ADR+
saxa
con ADR
(b)
RhoA
GAPDH
(kD)
250
150
100
75
50
37
25
20
ADR+
saxa
ADR+
saxa
con ADR
con ADR
Additional File 2
